# Eye and Systemic Disease Management Changes After Teleophthalmology Screening in Primary Care: Retrospective Cross-Sectional Pilot Study of 200 Consecutive Patients

**DOI:** 10.2196/81918

**Published:** 2025-10-24

**Authors:** Alexander Pinhas, Boris Pinhas, Egor Dmitruk, Stella Pinhas

**Affiliations:** 1 Department of Ophthalmology, Icahn School of Medicine at Mount Sinai, 1 Gustave L. Levy Place, New York, NY, 10029, United States, 1 9177108830; 2 myeye.healthcare LLC, Forest Hills, NY, United States; 3 Retina & Ophthalmology Services of New York PLLC, Elmhurst, NY, United States; 4 Internal Medicine & Geriatrics PLLC, Elmhurst, NY, United States

**Keywords:** teleophthalmology, asynchronous telemedicine, primary health care, diabetic retinopathy screening, age-related macular degeneration, interdisciplinary care

## Abstract

**Background:**

Undiagnosed ocular diseases and ocular complications from systemic diseases are common in primary care populations, and many can be detected through retinal imaging before symptoms develop. Asynchronous store-and-forward teleophthalmology offers a scalable way to integrate eye screening into primary care, yet its broader impact beyond diabetes and diabetic retinopathy detection remains underexplored.

**Objective:**

This study evaluated the outcomes of asynchronous store-and-forward teleophthalmology screening in a primary care clinic, including detection and triage of ocular conditions and subsequent changes in eye and systemic management.

**Methods:**

This was a retrospective cross-sectional analysis of the first 200 patients screened in a single primary care clinic in Elmhurst, New York, between January and May 2025. Each patient underwent nonmydriatic external and posterior eye imaging, which was reviewed by a remote reading eye clinician. Reports included eye findings, triage decisions (routine monitoring vs in-person referral), and management recommendations. Subsequent changes in care were extracted from primary care and in-person specialist consult notes.

**Results:**

Of 200 patients (mean age 62.1, SD 19.0, range 11‐100 years), 71.5% (143/200, 95% CI 64.9-77.3) had positive eye findings, and 40% (80/200, 95% CI 33.5‐46.9) were referred for in-person eye examinations. Only 8.8% (7/80, 95% CI 4.3-17.0) of referrals were for diabetic retinopathy; most were for glaucoma suspects, age-related macular degeneration, cataracts, and other eye diseases. Image quality was high, with 98.2% (390/397, 95% CI 96.4-99.1) of fundus images being at least partially adequate. Of the 32 patients with documented in-person eye follow-up, 87.5% (28/32) of evaluations confirmed the screening findings. Eye management changes were initiated in 11 patients, whereas systemic management changes occurred in 70 patients, including new prescriptions for Age-Related Eye Disease Study 2 supplements, antihypertensives, diabetes medications, and lipid-lowering agents.

**Conclusions:**

Asynchronous teleophthalmology screening in a primary care setting effectively identified both ocular diseases and ocular complications from systemic diseases, leading to meaningful changes in eye and systemic management. The low rate of diabetic retinopathy among referrals highlights the broader diagnostic value of retinal imaging beyond diabetes management. This care model offers a scalable, high-yield strategy for proactive disease detection and interdisciplinary intervention at the primary care level.

## Introduction

### Background

The US population is steadily growing and aging, with projections rising from 333 million in 2020 to 355 million by 2030. Adults aged 65 and older are expected to comprise 20% of the population by then, up from 17% in 2020 [1]. As a result, the prevalence of chronic medical conditions, including hypertension, diabetes mellitus, dyslipidemia, and heart disease, is projected to reach 171 million [2], while chronic eye diseases such as cataracts, diabetic retinopathy, glaucoma, and age-related macular degeneration are expected to affect 58 million individuals by 2030 [3]. In response to these trends, the CDC has called for a shift from reactive care to proactive, preventive healthcare [4]. This underscores the urgent need for scalable, cost-effective strategies for early detection, triage, and intervention in both systemic and ocular disease.

As a myriad of systemic diseases, including common cardiovascular and neurodegenerative disorders, manifest in the eyes, ocular biomarkers have long been proposed as noninvasive tools for detecting both ocular and systemic conditions [5]. Landmark studies such as the Diabetes Control and Complications Trial, the UK Prospective Diabetes Study, and the UK Prospective Diabetes Study-Hypertension in Diabetes Study demonstrated this potential, using serial color fundus photography to show that intensive glycemic and blood pressure control reduces microvascular complications in diabetes [6-8]. In 2001, EyePACS launched one of the earliest asynchronous store-and-forward teleophthalmology programs, enabling non–eye care settings to capture and upload fundus images for remote interpretation of diabetic retinopathy [9]. To support this model, the Centers for Medicare & Medicaid Services (CMS) and the American Medical Association introduced Current Procedural Terminology (CPT) codes 92227 and 92228 in 2011 for remote diabetic retinopathy screening in primary care. The National Committee for Quality Assurance Healthcare Effectiveness Data and Information Set also recognized nonmydriatic fundus photography that covers the macula and optic nerve as an acceptable form of eye examination in diabetics. However, early adoption was limited, likely because of insufficient imaging infrastructure and the lack of widespread, integrated telehealth ecosystems.

The ideas of remote eye screening and oculomics (the study of ocular biomarkers for systemic disease) have recently embarked on a renaissance because of advances in smaller, automated, and more affordable eye imaging technologies, such as color fundus cameras, alongside developments in data science, cloud computing, and artificial intelligence (AI) [10]. Between 2018 and 2024, 3 autonomous AI algorithms (ie, IDx-DR, EyeArt, and AEYE-DS) capable of diabetic retinopathy screening via color fundus photography without clinician input received Food and Drug Administration (FDA) approval [11-16]. In response, CMS approved CPT code 92229 in 2021, covering point-of-care AI-based retinal imaging. Despite this, real-world adoption has been limited. By 2023, the use of CPT 92229 remained low (59 per 100,000), significantly trailing the traditional remote interpretation codes 92227 and 92228 (156 per 100,000), which have nearly tripled in usage since 2021. Still, even these remain far below conventional in-clinic fundus photography (CPT 92250), which had a 6 times higher usage rate of 897 per 100,000 in 2023 [17].

Adoption of autonomous AI for diabetic retinopathy screening faces several barriers. Economically, the appeal is limited because only approximately 15% to 20% of the primary care population has diabetes [18,19], making it difficult for non–eye clinics to justify investing in imaging equipment solely for this purpose [20]. The expansion of indications for AI beyond diabetic retinopathy screening is pushing forward, with glaucoma, age-related macular degeneration, retinopathy of prematurity, and ocular oncology identified as priority areas [21]. Recent studies also highlight the growing potential of retinal imaging combined with AI to detect and risk stratify a range of systemic chronic conditions through the eye, including hypertension [22], diabetes mellitus [23], metabolic syndrome [24], atherosclerotic cardiovascular disease [25,26], chronic kidney disease [27], and neurodegenerative disorders such as Alzheimer dementia [28-30]. However, each new indication requires separate FDA approval tied to specific devices, involving time- and cost-intensive clinical trials. In addition, fully autonomous algorithms often yield higher false-positive rates when no human oversight is involved, potentially increasing unnecessary referrals and placing further strain on already limited eye care resources [31,32].

Between 2021 and 2022, CMS expanded the range of International Classification of Diseases, 10th Revision, codes eligible for CPT codes 92227 and 92228, reflecting growing evidence that tele-ophthalmology can detect a variety of retinal diseases beyond diabetic retinopathy. The COVID-19 pandemic further accelerated telehealth adoption, prompting rapid regulatory shifts, expanded reimbursement, and widespread integration of remote care technologies [33]. Other CPT codes such as 99451, which reimburse for written virtual interprofessional consultations, were allowed to be used alongside CPT code 92228 to support human-led virtual eye care coordination [34]. These policy shifts likely contributed to the observed 3-fold increase in the usage of CPT codes 92227 and 92228 since 2021.

The asynchronous store-and-forward model, where images are captured in non–eye clinics, uploaded to the cloud, and interpreted by remote eye clinicians, has proven both clinically effective and economically viable [34,35]. Unlike autonomous AI, this model allows broader screening across ocular pathologies and may yield higher specificity, reducing unnecessary referrals. Emerging research suggests that co-piloting, or combining assistive AI with human oversight, offers the most balanced performance in sensitivity, specificity, and cost-effectiveness [32]. As the field evolves, the optimal future may lie in hybrid models that integrate both approaches. For now, asynchronous human-led screening provides a practical and scalable solution.

### Objectives

This study aimed to evaluate the outcomes of a pilot asynchronous teleophthalmology program in a primary care setting, assessing the prevalence of ocular and systemic findings beyond diabetic retinopathy. In addition, it examined how screening results influenced subsequent eye and systemic disease management.

## Methods

### Overview

**Figure 1.**
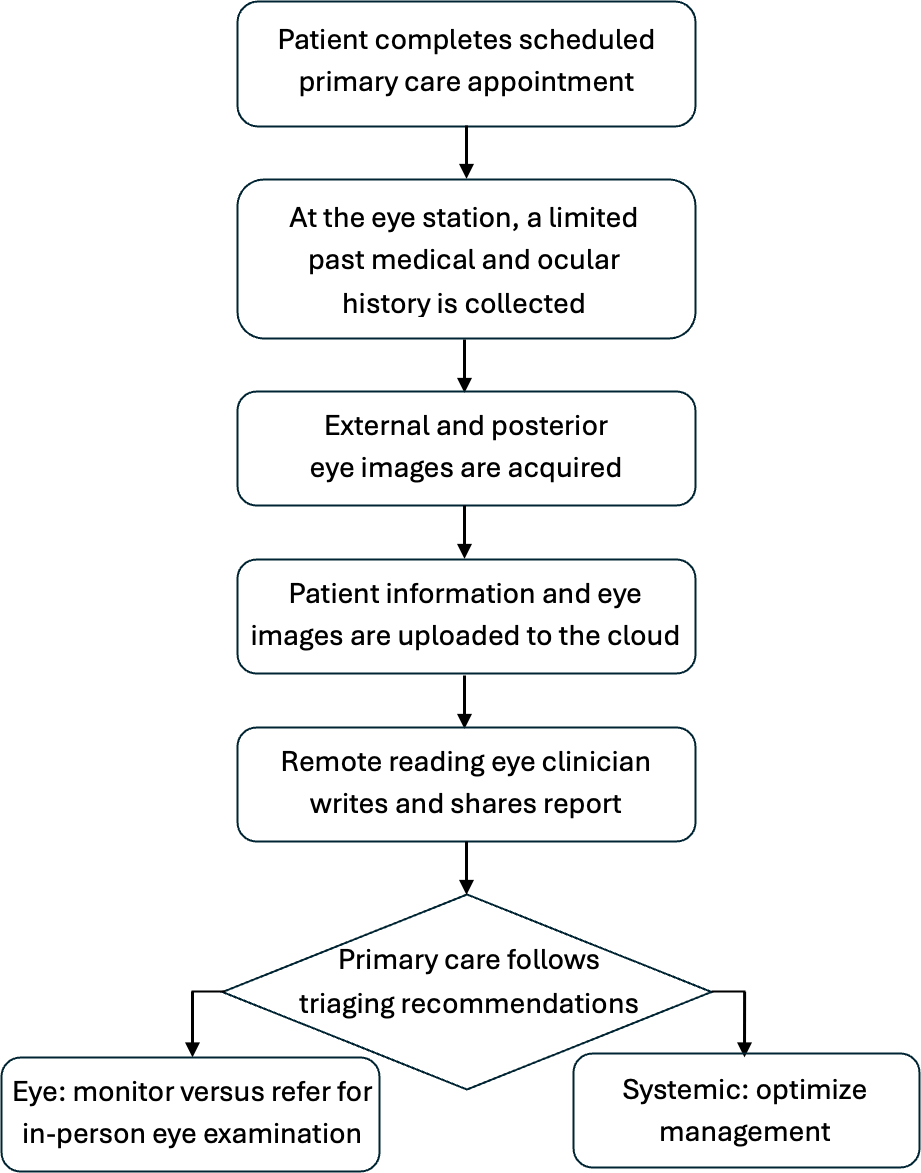
Asynchronous store-and-forward tele-ophthalmology workflow diagram.

This was a retrospective, cross-sectional, descriptive pilot study reviewing the first 200 consecutive patients enrolled in a teleophthalmology program at a single primary care clinic (Internal Medicine & Geriatrics PLLC, Elmhurst, NY) between January and May 2025 (Figure 1). Reporting follows Strengthening the Reporting of Observational Studies in Epidemiology, and a completed checklist with page references is provided (Checklist 1). Six weeks were allowed to pass after the 200th virtual eye consult report was received, before performing the chart review. Data sources included virtual eye consult reports by a single remote reading eye clinician, as well as follow-up documentation in non–eye clinician progress notes and in-person eye consult records. No additional inclusion or exclusion criteria were applied.

### Ethical Considerations

This study was reviewed and granted exempt status by the Castle Institutional Review Board (IRB), as only deidentified patient data were analyzed. Castle IRB is an independent IRB, which has full accreditation with the Association for the Accreditation of Human Research Protection Programs and is registered with both the US Office for Human Research Protections and the FDA as IRB Organization number: IORG0010151 and IRB Registration number: IRB00012054. As deidentified patient data were used in this retrospective review, informed consent was not required, and no compensation was provided to patients. The study adhered to the tenets of the Declaration of Helsinki and all applicable Health Insurance Portability and Accountability Act regulations. It was ensured that no identification of individual patients in any images of the manuscript is possible.

### Variables

 Descriptive variables in this study included age (years, continuous), sex (female, male), race or ethnicity (White, Asian, Hispanic, Black), past medical history (diabetes mellitus, hypertension, dyslipidemia, obesity), self-reported past ocular history (cataract, diabetic retinopathy, glaucoma, age-related macular degeneration), and active visual symptoms at screening (decreased vision, floaters, or flashes).

 Prespecified key outcomes with 95% Wilson CIs included image quality adequacy, positive eye findings, referral for in-person eye examination, distribution of referable eye diagnoses, and systemic management changes initiated. Other descriptive outcomes included in-person eye referral completion, screening concordance with in-person eye consultations, in-person non–eye referral completion, eye management changes initiated, and patient compliance with new plans. Number needed to screen (NNS=1/proportion) for select outcomes were calculated, including referral for in-person eye examination and systemic management changes initiated. We did not perform adjusted or interaction analyses because this pilot was descriptive and not designed to test associations.

### Sample Size Rationale

The sample size was predefined to ensure acceptable descriptive precision for key proportions (eg, positive screens and referral rate) with an acceptable margin of error between 5% and 10% at 95% CI. Using n=[z^2^p(1−p)]/E^2^, where n is the sample size, z is 1.96 at 95% CI, and the conservative anticipated proportion p is 0.50, our target margin of error (E) between 5% and 10% implied a necessary sample size between 97 (for E=10%) and 385 (for E=5%). The sample size of 200 was chosen as a pragmatic midpoint.

### Virtual Eye Consult Reports

All virtual eye consults in this pilot were interpreted by 1 remote eye clinician, a board-certified ophthalmologist fellowship trained in medical and surgical diseases of the vitreous and retina. The reader had 4 years of clinical experience, with routine use of color fundus photography in practice. The reader applied prespecified diagnostic (International Clinical Diabetic Retinopathy [ICDR] for diabetic retinopathy, Wong–Mitchell for hypertensive retinopathy, Age-Related Eye Disease Study [AREDS] for age-related macular degeneration, and standard optic nerve features for glaucoma suspects) and referral criteria as described in the Referable Eye Disease Definitions section. The reader had access to basic demographics and past medical and ocular history embedded in the consult request. The reader was unaware of later in-person eye examination findings.

The virtual eye consult reports included demographic information (age, sex, race/ethnicity), past medical history (diabetes mellitus, hypertension, dyslipidemia, obesity), self-reported past ocular history (cataracts, diabetic retinopathy, glaucoma, age-related macular degeneration), and active visual symptoms at screening (decreased vision, floaters, or flashes). Each report featured a photo analysis section assessing image quality and documenting image findings based on 1 nonmydriatic 45° center-nasal single-field color fundus photograph (centered between the fovea and the disc center) and 1 external eye photograph per eye (DRSplus; iCare USA Inc, Raleigh, North Carolina). The reports concluded with an assessment and plan, including a problem list, management recommendations for the primary care clinician, and guidance on whether in-person eye or systemic referrals were warranted.

### Image Quality Review

The virtual eye consult reports included an assessment of image quality, classified as adequate, partially adequate, or inadequate. Grading was subjective and followed criteria adapted from prior studies [36]. Quality consideration considered four main domains: sharpness, illumination, field of view, and artifacts. Sharpness was assessed based on image focus. For external images, this included iris patterns and eyelash clarity, whereas for posterior images, it included foveal detail, tertiary retinal vessel, and cup and disc margin clarity. Illumination was deemed suboptimal if dark or overexposed areas interfered with visualization of standard anatomical features. Field of view was considered adequate when both the foveal center and the entire optic nerve head were within the field of imaging. Artifacts included dust spots, sensor defects, or obstruction by lids and lashes that impaired image interpretation.

### Referable Eye Disease Definitions

#### Overview

The remote reading eye clinician used standardized criteria to determine whether a patient should be referred for an in-person eye examination. Definitions for each condition were as follows.

#### Cataracts

External eye color photographs were used to screen for cataracts, although evaluation was limited by nonmydriatic imaging. As a result, only more advanced cataracts, typically dense, brunescent, or white, were reliably detected and considered referable.

#### Diabetic Retinopathy

Referable diabetic retinopathy was defined as more-than-mild nonproliferative diabetic retinopathy, which is defined by the ICDR scale as the presence of signs beyond microaneurysms [37]. This includes dot-blot hemorrhages, exudates, venous beading, intraretinal microvascular abnormalities, neovascularization, preretinal hemorrhages, and macular edema. Macular edema was defined as exudates or apparent thickening within one-disc diameter of the fovea.

#### Hypertensive Retinopathy

Referable hypertensive retinopathy was defined as more-than-mild hypertensive retinopathy, which is defined by the Wong and Mitchell scale as the presence of signs beyond arteriolar narrowing, copper wiring, and arteriovenous nicking [38]. This includes dot-blot hemorrhages, flame-shaped hemorrhages, microaneurysms, cotton-wool spots, hard exudates, and optic disc swelling.

#### Glaucoma Suspect

As glaucoma grading requires imaging and testing beyond fundus photography, including intraocular pressure measurement, visual fields, and optical coherence tomography retinal nerve fiber measurements, screening with fundus photography was only able to detect glaucoma suspects (unless the patient self-reported a history of glaucoma).

Glaucoma suspect, or referable possible glaucoma, was defined as a large cup-to-disc ratio (a ratio of vertical cup to disc diameter of 0.6 or greater), asymmetry in cup-to-disc ratio between eyes (a difference >0.2), neuroretinal rim thinning or notching, optic disc pallor, and optic disc hemorrhages (eg, Drance hemorrhages) [39].

#### Age-Related Macular Degeneration

Referable age-related macular degeneration was defined using the AREDS grading system as the presence of more-than-mild age-related macular degeneration, including at least 20 medium-sized drusen (63‐124 microns), at least one large drusen (≥125 microns), pigmentary changes associated with large drusen, central or noncentral geographic atrophy, and neovascular membranes [40].

#### Other Conditions

 For other pathologies, clinical judgment guided referral decisions. Patients were also referred if they reported concerning active visual symptoms, such as decreased vision, floaters, or flashes.

#### Referrals Based on Image Quality

If image quality was deemed partially adequate or inadequate, and referable disease could not be confidently ruled out, patients were referred for in-person eye evaluation.

### Primary Care Clinician Progress Notes and In-Person Consult Notes

Progress notes from the primary care clinician and notes from in-person eye and noneye consultations were reviewed to determine whether an eye referral was made and completed, whether a noneye referral was made and completed, whether the initial remote report findings were confirmed by the referring clinician or consulting providers, whether changes were made to eye management, whether changes were made to systemic management, and whether the patient was compliant with recommended management changes.

### Bias and Data Quality

We took several steps to limit common biases in this retrospective cross-sectional pilot. To minimize selection bias, we used consecutive sampling of patients with no post hoc exclusions. We also used a fixed abstraction window, beginning chart review 6 weeks after imaging the 200th patient. To minimize classification bias, standardized protocols were used to acquire images, grade image quality, and grade ocular findings and referability. When disease could not be ruled out because of poor image quality, patients were referred, favoring sensitivity over missed disease. To minimize observer bias and incorporation bias, the remote reader rendered reports before any in-person eye referral outcomes existed.

To ensure data abstraction quality, we used a structured abstraction template with explicit variable definitions and maintained a data provenance log. Summary counts and CIs were independently checked by a second author. Effort was taken not to count missing data as an outcome verification. Concordance with in-person assessments was calculated only where consultant notes were available. Referral completion and compliance were counted as completed, not completed, or unknown rather than treating missing data as negative. As this pilot was descriptive with small event counts and incomplete follow-up for some referrals, we did not perform adjusted or interaction analyses, reducing the risk of overfitting or spurious inference.

## Results

### Demographics and Systemic Disease Prevalence

The study included 200 patients, with a mean age of 62.1 years (SD 19.0; range: 11‐100 years). Of these, 122 (61%) were female, and 78 (39%) were male. Self-identified race or ethnicity was as follows: 140 (70%) White, 31 (15.5%) Asian, 26 (13%) Hispanic, and 3 (1.5%) Black. Of the 200 patients, 52 (26%) had type 2 diabetes mellitus, 97 (48.5%) had hypertension, 146 (73%) had dyslipidemia, and 35 (17.5%) were classified as obese (Table 1). Self-reported past ocular histories included 18 (9%) patients reporting a history of cataract, 38 (19%) patients reporting having had cataract surgery (this group was mutually exclusive from the history of cataract group), 3 (1.5%) patients reporting a history of diabetic retinopathy, 11 (5.5%) patients reporting a history of glaucoma, 4 (2%) patients reporting a history of age-related macular degeneration, and 13 (6.5%) patients reporting having some active visual symptoms.

**Table 1.** Demographics and systemic disease prevalence.

Variable	Results (N=200 patients)
Age (years)	
Mean (SD)	62.1 (19.0)
Range	11‐100
Sex, n (%)	
Female	122 (61.0)
Male	78 (39.0)
Race/ethnicity, n (%)	
White	140 (70.0)
Asian	31 (15.5)
Hispanic	26 (13.0)
Black	3 (1.5)
Past medical history, n (%)	
Diabetes mellitus	52 (26.0)
Hypertension	97 (48.5)
Dyslipidemia	146 (73.0)
Obesity	35 (17.5)

### Imageability Metrics

**Figure 2.**
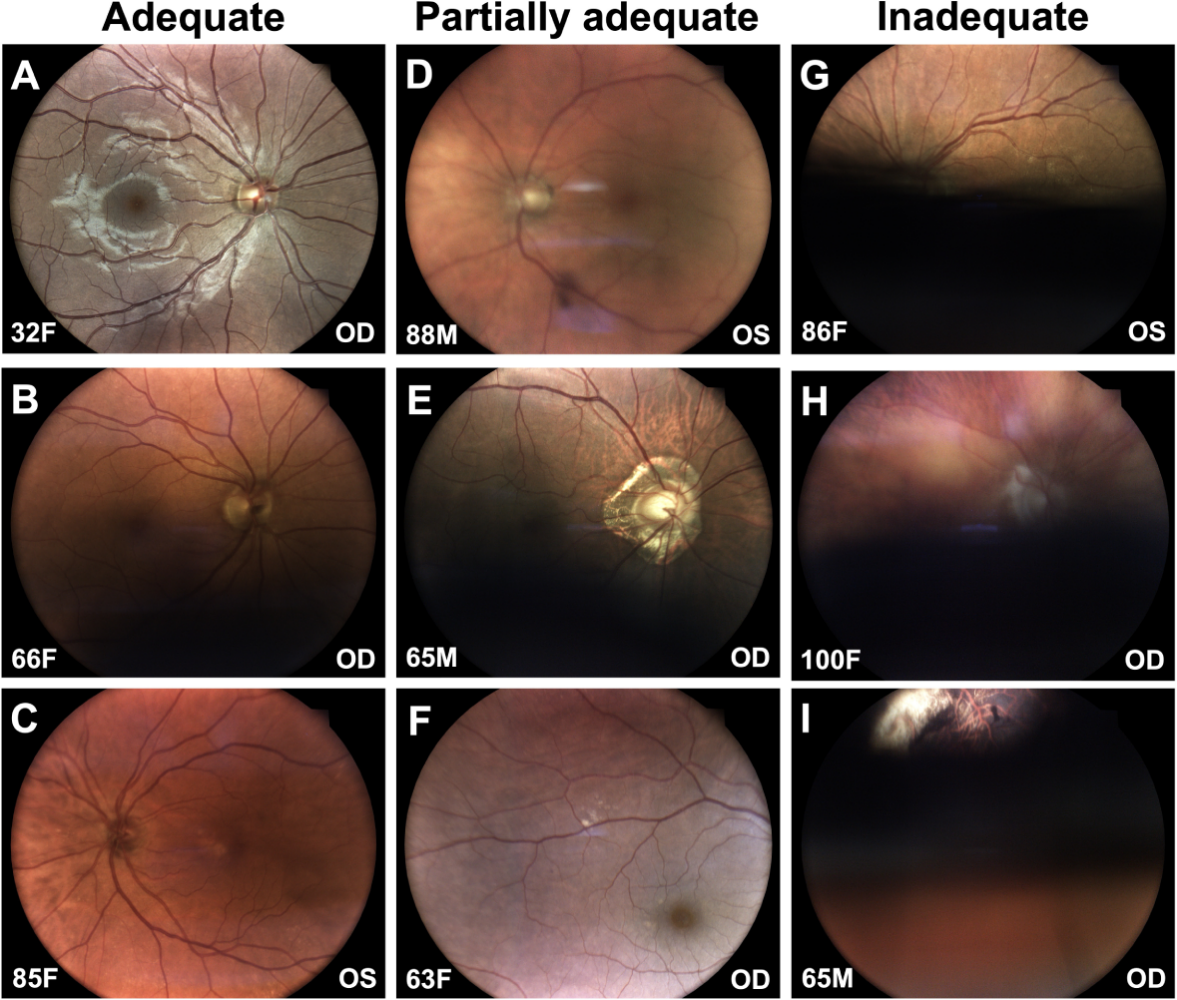
Image quality assessment examples of posterior eye images. The first column showcases adequate images, the second column showcases partially adequate images, and the third column showcases inadequate images. Panel A shows the average image, without any defects. Panel B shows an illumination defect with an inferior shadow that is not too dark, still allowing discernment of blood vessels in the area. Panel C shows a sharpness defect with mild blurriness that still allows discernment of anatomic features. Panel D shows a sharpness defect with moderate blurriness that makes it more difficult to discern anatomic features. Panel E shows an illumination defect with an inferior shadow that is darker, making discernment of blood vessels in the area difficult. Panel F shows a field of view defect, where the fovea is visible but the optic nerve head is not visible. Panel G shows an illumination defect with an inferior shadow that covers visibility of the fovea and optic nerve head. Panel H shows an inferior illumination defect and a superior sharpness defect. Panel I shows an artifact defect allowing only a small portion of the posterior segment to be visualized. The bottom of each image is labeled with age and sex of the patient, and eye laterality (M=male; F=female; OD=right eye; OS=left eye).

Strict imageability was defined as the proportion of images subjectively graded as adequate out of the total number of images assessed. Lenient imageability included both adequate and partially adequate images. For external eye images, 400 images were expected (2 per eye×200 patients), but 3 were missing due to monocular status. Of the 397 images obtained, 396 were adequate, and 1 was partially adequate, resulting in strict imageability of 99.7% (95% CI 98.6-100) and lenient imageability of 100% (95% CI 99.0-100). For posterior eye images, 400 were expected, with the same 3 missing due to monocular status. Of the 397 images obtained, 342 were adequate, 48 were partially adequate, and 7 were inadequate. This resulted in a strict imageability of 86.1% (95% CI 82.4-89.2) and a lenient imageability of 98.2% (95% CI 96.4-99.1; Table 2; Figure 2).

**Table 2.** Eye screening and triaging key outcomes.

Outcome	Images (N1=397) and patients (N2=200), n (%; 95% CI).	NNS^a^ (95% CI)
Posterior image lenient adequacy	390 (98.2; 96.4-99.1)	-^b^
Positive eye findings	143 (71.5; 64.9-77.3)	-
Referred for in-person eye examination	80 (40.0; 33.5-46.9)	2.50 (2.13-2.99)
Positive eye findings not referred	63 (31.5; 25.5-38.2)	-
Systemic management changes	70 (35.0; 28.7-41.8)	2.86 (2.39-3.48)

a NNS: number needed to screen.

b -: not applicable.

### Eye Screening and Triaging Outcomes

The following percentages and 95% CIs are presented relative to all 200 patients screened. Of the 200 patients screened, 57 (28.5%; 95% CI 22.7-35.1) had negative eye screens, whereas 143 (71.5%; 95% CI 64.9-77.3) had positive findings (Table 2). Among those with positive findings, 76 (38%; 95% CI 31.6-44.9) had 1 finding, 48 (24.0%; 95% CI 18.6-30.4) had 2 findings, and 19 (9.5%; 95% CI 6.2%-14.4%) had 3 findings. A total of 80 (40%) patients (95% CI 33.5‐46.9) had referable disease and were referred for in-person eye examinations, corresponding to screening 2.5 patients per referral (NNS 2.50; 95% CI 2.13-2.99; Table 2). The remaining 63 (31.5%) patients (95% CI 25.5-38.2) with positive findings that did not meet the threshold for referral were managed by the primary care clinician and scheduled for annual remote monitoring.

**Figure 3.**
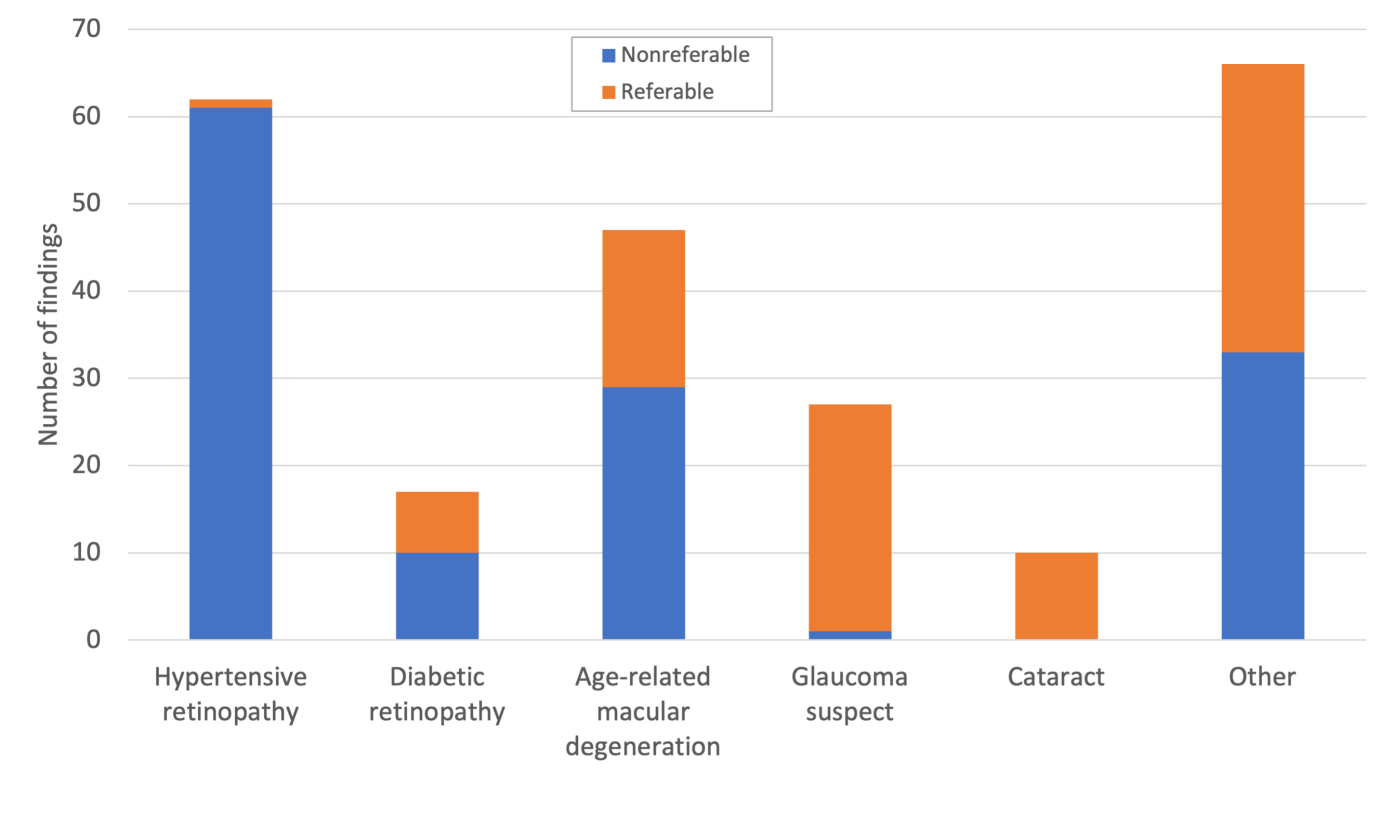
Distribution of eye findings and referability.

There were 62 cases (31%; 95% CI 25.0-37.7) of hypertensive retinopathy (1 referable, 0.5%; 95% CI 0.1-2.8), 17 cases (8.5%; 95% CI 5.4-13.2) of diabetic retinopathy (7 referable, 3.5%; 95% CI 1.7-7.0), 47 cases (23.5%; 95% CI 18.2-29.8) of age-related macular degeneration (18 referable, 9.0%; 95% CI 5.8-13.8), 27 cases (13.5%; 95% CI 9.4-18.9) of glaucoma suspect (26 referable, 13.0%; 95% CI 9.0-18.4), 10 cases of referable cataract (5.0%; 95% CI 2.7-9.0), and 66 cases (33.0%; 95% CI 26.9-39.8) of other findings (33 referable, 16.5%; 95% CI 12.0-22.3; Figures3  4). Of note, these categories were not mutually exclusive, so percentages sum to more than 100%. Other findings included dry eye, pterygium, xanthelasma, age-related ptosis, ectropion, iris nevus, retinal vascular occlusion, long-term hydroxychloroquine use, choroidal nevus, myopic degeneration, epiretinal membrane, vascular changes in nondiabetics/nonhypertensives, retinal aneurysm, blurring of disc margins, nonglaucomatous optic neuropathy, complaints of subjective visual disturbances (including floaters, flashes, and decreased vision), and low vision or monocular status. Remote eye clinician findings did not align with 13 self-reported cataracts, likely due to the difficulty assessing for cataract in the nonmydriatic setting.

Notably, only 7 (8.8%) of the 80 referrals (95% CI 4.3-17.0) were for diabetic retinopathy. Comparing remote reading eye clinician findings to self-reported past ocular histories, patients were unaware of their eye conditions in 193 (84.3%) of the 229 unique findings (95% CI 79.0-88.4), highlighting the value of proactive screening.

**Figure 4.**
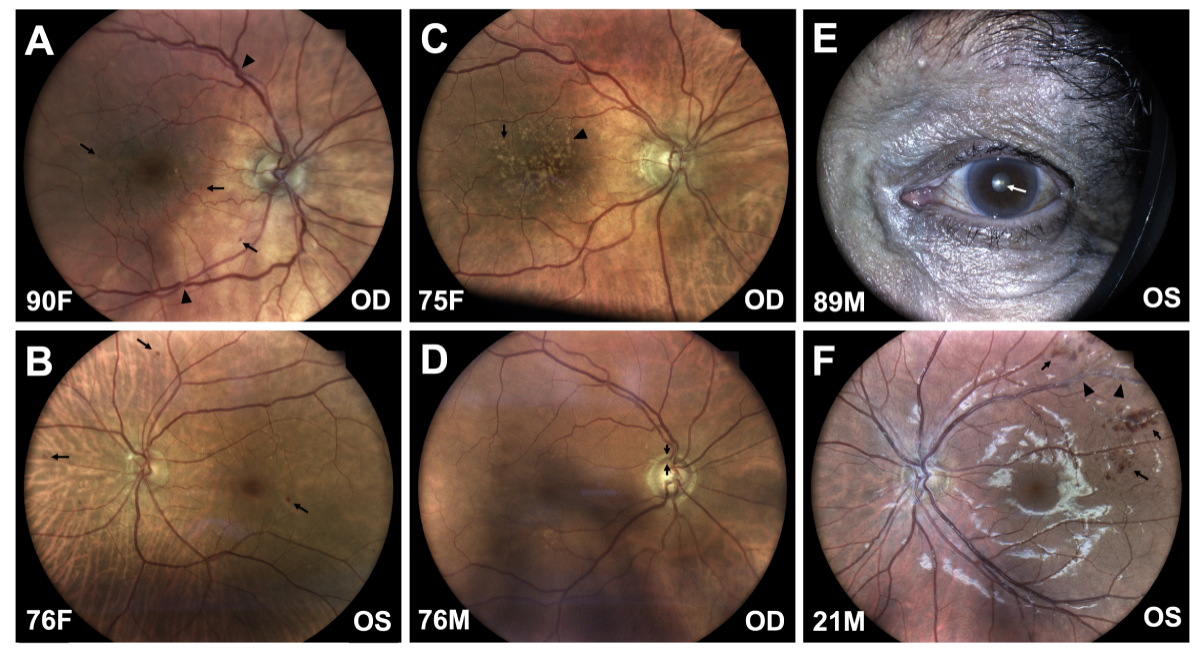
Referable eye disease examples. (A) Moderate hypertensive retinopathy: A 90-year-old female with hypertension and dyslipidemia was found to have diffuse arteriolar attenuation with arteriovenous nicking (arrowheads), increased venous tortuosity, and multiple dot-and-blot intraretinal hemorrhages (arrows). (B) Moderate nonproliferative diabetic retinopathy: A 76-year-old female with diabetes mellitus, hypertension, dyslipidemia, and obesity was found to have scattered dot-and-blot intraretinal hemorrhages (arrows). (C) Intermediate age-related macular degeneration: A 75-year-old female with hypertension and dyslipidemia was found to have numerous medium-sized (arrow) and large-sized (arrowhead) drusen. (D) Glaucoma suspect: A 76-year-old male was found to have asymmetric cupping of his optic nerve head, right greater than left (arrows demarcate the thinned optic nerve rim). He was subsequently confirmed to have glaucoma at his in-person eye visit. (E) Cataract: An 89-year-old male was found to have brunescence of his lens that was visible even without pupillary dilation (arrow). Cataract was subsequently confirmed at his in-person eye visit, and cataract surgery is being planned. (F) A 21-year-old otherwise healthy male was found to have dot-and-blot intraretinal hemorrhages (arrows) and perivenular sheathing (arrowheads). He was subsequently diagnosed with idiopathic retinal vasculitis at his in-person eye visit and was started on systemic immune modulators. The bottom of each image is labeled with age and sex of the patient and eye laterality (M=male; F=female; OD=right eye; OS=left eye).

### Referrals and Changes in Management

#### In-Person Eye Consults Triggered From Eye Screening Findings

The remote reading eye clinician recommended in-person eye examinations for 80 of the first 200 patients based on findings of referable eye disease. The primary care clinician adhered to all recommendations, initiating referrals for each of the 80 patients. Referral communication varied and included telephone calls, face-to-face discussions, and written prescriptions. Referrals were either general or directed to specific eye care providers. Nineteen (23.8%) referrals were nonspecific, advising patients to see an eye clinician (optometry or ophthalmology). Sixty-one (76.3%) referrals were specialty specific, with 15 (18.8%) to comprehensive ophthalmology, 42 (52.5%) to retina specialists, 12 (15%) to glaucoma specialists, 1 (1.3%) to uveitis, 1 (1.3%) to oculoplastics, and 1 (1.3%) to low vision optometry. Of the 80 patients, 71 (88.8%) patients were referred to 1 eye care specialty, 7 (8.8%) patients were referred to 2 eye specialties, and 2 (2.5%) patients were referred to 3 eye specialties.

Of the 80 patients who were recommended for in-person eye evaluations, the records indicated that 35 patients (43.8%) reported completing at least one in-person eye visit, 12 patients (15%) reported not completing any in-person eye visit, and 44 patients (55%) had no documentation of compliance (there was no in-person eye consult note or a patient report in the primary care clinic chart). Among the 35 patients who reported completing a visit, 32 in-person eye consult reports were available for review. Of these 32 consult reports, 28 reports (87.5%) confirmed agreement with the findings of the virtual eye consult, and 4 reports (12%) documented disagreement. In these 32 reviewed consults, 11 patients (34.4%) had documented new eye management changes, which included 4 cases of closer observation or additional workup, 3 recommendations for topical eye drops, 2 instructions to perform Amsler grid testing at home, 1 planned glaucoma laser procedure, and 1 planned cataract surgery. Regarding compliance with these new management plans, 8 patients (72.7%) were documented as compliant, 1 patient (9.1%) was documented as noncompliant, and 2 patients (18.2%) had no compliance documentation. Given the small denominator, these figures are descriptive, and CIs are not presented.

#### In-Person Non–Eye Consults Triggered From Eye Screening Findings

On the basis of virtual eye consult report findings and recommendations, the primary care clinician initiated 7 in-person non–eye specialist consults. Of these, 6 consults were completed, and 1 consult had no documentation of completion (ie, no consult note or compliance record in the primary care clinic chart). In-person non–eye consults included 3 endocrinology consults for improved glycemic control, 2 cardiology consults (1 for a patient with suspected central retinal vein occlusion to improve blood pressure control and assess for carotid artery stenosis and 1 for a patient with retinal aneurysm and concurrent left arm pain), 2 rheumatology consults (1 for the central retinal vein occlusion patient to evaluate for a hypercoagulable state and 1 for a patient with idiopathic retinal vasculitis), and 1 pediatric and 1 genetic counseling consult for an 11-year-old patient with retinal tortuosity to evaluate for connective tissue disease. The central retinal vein occlusion patient had no significant carotid artery stenosis and a negative hypercoagulable workup and was started on aspirin. The patient with the retinal aneurysm and arm pain was found to have a bridging left anterior descending coronary artery and was started on metoprolol. The pediatric patient was found not to have a connective tissue disorder.

#### Systemic Management Changes Triggered by Eye Imaging Findings

On the basis of the findings from eye imaging, systemic management changes were made for 70 patients (35%; 95% CI 28.7‐41.8), corresponding to screening ~3 patients per systemic management change (NNS 2.86; 95% CI 2.39-3.48; Table 2). These changes included 34 initiations of oral AREDS2 supplements (17%); 17 hypertension optimizations (8.5%), involving adjustments or initiations of medications, such as minoxidil, losartan, enalapril, carvedilol, metoprolol, hydrochlorothiazide, and spironolactone; 11 diabetes mellitus optimizations (5.5%), involving semaglutide, tirzepatide, metformin, insulin, and empagliflozin; 7 dyslipidemia optimizations (3.5%) primarily involving statins; 8 weight optimizations (4%) involving semaglutide and tirzepatide; 1 initiation on aspirin; 1 dietary intervention; and 1 initiation of mycophenolate mofetil. Some patients received more than 1 systemic intervention. In terms of compliance, 41 (58.6%) of these 70 patients were documented as compliant, 12 patients (17.1%) were documented as noncompliant, and 17 patients (24.3%) had no available compliance documentation.

## Discussion

### Principal Findings and Relevance

This retrospective cross-sectional analysis of the first 200 patients screened via asynchronous teleophthalmology at a primary care clinic provides early real-world insights into the feasibility, diagnostic yield, and triage outcomes of integrating remote eye screening into non–eye clinical settings. The findings support the study’s central objective to evaluate whether asynchronous store-and-forward telehealth technology can identify a meaningful prevalence of both ocular disease and systemic manifestations through eye imaging in a primary care setting and appropriately triage patients for either continued monitoring or in-person evaluation. The findings also support the idea that teleophthalmology screening results can guide changes in eye and systemic disease management.

In our study, 40% of the total cohort was referred for in-person eye examinations based on referable pathology, indicating that every 2.5 patients screened prompted 1 in-person eye referral. This referral rate is consistent with prior teleophthalmology studies, which have reported referral rates ranging from approximately 20% to 48%, depending on the population screened and the range of ocular pathologies assessed [41]. Notably, only 8.8% of our referrals were for diabetic retinopathy, reinforcing the central hypothesis that the ocular disease burden in primary care populations extends far beyond diabetes-related pathology. A mobile teleophthalmology screening program in New York City reported screening 957 individuals in a low socioeconomic minority community, finding that only 29 (3%) individuals had diabetic retinopathy, whereas 305 (32%) had glaucoma, 124 (13%) had cataract, 9 (1%) had macular degeneration, and 97 (10%) had other eye diseases [42]. The higher cumulative prevalence of findings related to eye diseases other than diabetic retinopathy, including hypertensive retinopathy, age-related macular degeneration, glaucoma suspicion, and cataract, underscores the broader potential of eye screening programs as a general tool for chronic disease surveillance, not solely limited to diabetes care.

In our study, patients were unaware of their eye conditions 84.3% of the time. Prior literature aligns with this finding, showing substantial patient unawareness of having eye diseases, with diabetic retinopathy at a ~70% unawareness rate [43], age-related macular degeneration at a >80% unawareness rate [44], and open-angle glaucoma at a ~50% unawareness rate [45]. These statistics underscore the value of proactive, point-of-care eye screening and patient education in primary care settings.

The strong imageability metrics, 98.2% lenient and 86.1% strict adequacy for posterior images, were consistent with those reported in prior literature [46] and confirm that high-quality, nonmydriatic imaging is achievable in routine primary care workflows using minimal training and without the need for pupil dilation. Follow-through from primary care clinicians on virtual consult recommendations was high, with all patients flagged for referral receiving the appropriate communication. Of those who kept their in-person eye appointments and had documentation available, a strong concordance rate was observed, indicating an appropriate clinical accuracy of this asynchronous model.

The relatively high proportion of patients undergoing systemic management changes based on ocular findings in our study underscores the close linkage between eye and systemic disease and that a significant portion of interventions for common eye diseases, such as diabetic retinopathy, hypertensive retinopathy, and age-related macular degeneration, is systemic rather than purely ocular. While AI-based cardiovascular risk calculators that incorporate retinal imaging are not yet FDA-approved in the United States, primary care clinicians can still apply clinical gestalt to interpret retinal findings when managing “gray zone” patients. This includes optimizing blood pressure, lipid levels, and glycemic control based on suggestive ocular markers. In the absence of validated AI tools, this human-guided approach remains a practical and immediately implementable strategy for enhancing chronic disease management.

Nearly half of the systemic management changes involved initiating oral AREDS2 supplements, which contain vitamin C (500 mg), vitamin E (400 IU), zinc (80 mg with 2 mg copper), lutein (10 mg), and zeaxanthin (2 mg). These supplements have been shown to reduce the risk of progression to advanced age-related macular degeneration by about 78 cases per 1000 people with intermediate (referable) age-related macular degeneration and 4 cases per 1000 with early (nonreferable) age-related macular degeneration [47-49]. Additional studies have reported improvements in visual acuity and contrast sensitivity in patients with early age–related macular degeneration taking AREDS2 [50].

With an estimated 30 million Americans affected by age-related macular degeneration, 25.6 million with early or intermediate forms, and 5.4 million with advanced disease [51], there is a critical need for early intervention. Furthermore, there is mounting evidence that a major pathophysiological component of age-related macular degeneration is vascular disease, and the presence of advanced age-related macular degeneration may serve as a biomarker for systemic vascular health [52]. Thus, primary care clinicians, when informed of their patients’ age-related macular degeneration status, have the potential to play a valuable role in early management, including through AREDS2 supplementation, dietary and lifestyle modifications, and systemic vascular health interventions. It should be considered that AREDS2 supplements are over the counter and are typically not covered by insurance.

### Limitations

Despite these encouraging findings, the study had several limitations. First, the retrospective nature and reliance on electronic chart review may have introduced information bias due to incomplete documentation, particularly regarding compliance with referrals or recommended management changes. A considerable proportion of referred patients had no documented follow-up status, which limited our ability to fully assess downstream clinical impact. A chart review was conducted only 6 weeks after the 200th patient was screened, which may not have allowed sufficient time for some of the later patients to complete in-person eye consultations and for those outcomes to be documented in the medical record. In addition, the absence of a control group limited causal inference, as systemic management changes may have been implemented without eye imaging information.

Furthermore, the study’s setting, a single clinic in Elmhurst, New York, may have introduced selection bias, given the specific demographic and socioeconomic context of the patient population. Most patients self-identified as White, which may not reflect broader urban or national diversity. Also, the geriatrics setting is enriched for older adults and higher chronic disease prevalence, limiting the generalizability of our findings to other primary care settings. For example, our clinic had a higher diabetes prevalence (26%) compared to reported averages in the primary care setting (15%‐20%).

While referability criteria were based on established grading systems, the interpretation of images and decision for referral was left to the discretion of a single reading eye clinician. A single-reader design may have introduced reader-level bias from the subjectivity of image grading and disease classification and may limit reproducibility. Furthermore, the magnitude of potential false positives or negatives, especially in cases graded as partially adequate, remains unknown without a systematic rereview of all cases by a second grader or adjudication panel. The impact of these biases was likely moderate; however, it should be acknowledged that both over- and under-referral could have occurred, affecting the downstream burden on specialty care or the risk of missed pathology. Other key limitations of this study involved limitations in digitizing the eye examination, including lack of dilation, lack of slit lamp visualization of anterior eye structures, and lack of peripheral retinal visualization outside of the single 45° field, all of which could have contributed to missed referable and vision-threatening pathology.

Interpretation of these results must be cautious, particularly in the context of emerging AI-based screening alternatives. While FDA-cleared autonomous AI tools for diabetic retinopathy offer scalability, their narrow indication, lower specificity, and limited adoption to date contrast with the broader diagnostic scope and nuanced triage possible via asynchronous human grading. Our findings support the view that human-led teleophthalmology remains a valuable, perhaps superior, solution in its current form, especially for complex, multipathology detection in high-risk populations. However, these results also align with growing evidence, suggesting a hybrid “clinician+AI” model may ultimately yield the best balance of efficiency, sensitivity, and specificity [32,53,54].

### Conclusions

This study provides early evidence that asynchronous teleophthalmology screening integrated into primary care settings can successfully identify a wide range of ocular and systemic diseases, with high patient imageability, diagnostic yield, and clinician and patient follow-through. The generalizability of this study is limited by the single-center design, yet the model itself is inherently scalable. The success of the imaging device used (a nonmydriatic fundus camera) and the asynchronous written communication between the remote reading eye clinician and the primary care clinician suggest that this approach could be feasibly replicated in other primary care settings, particularly those serving aging or chronically ill populations. While the model requires further validation through prospective, multicenter studies and longer-term outcomes tracking [55], its potential to expand access, reduce missed diagnoses, and enhance care coordination across disciplines is compelling. As health care continues to evolve toward proactive, interdisciplinary, and digitally-enabled care models, asynchronous eye screening at the frontlines of medicine deserves further investment and evaluation.

## Supplementary material

10.2196/81918 Checklist 1 STROBE checklist.

## Abbreviations:

AI: artificial intelligence
AREDS: Age-Related Eye Disease Study
CMS: Centers for Medicare & Medicaid Services
CPT: Current Procedural Terminology
FDA: Food and Drug Administration
ICDR: International Clinical Diabetic Retinopathy
IRB: institutional review board
